# Effect of Alkaloids from Nelumbinis Plumula against Insulin Resistance of High-Fat Diet-Induced Nonalcoholic Fatty Liver Disease in Mice

**DOI:** 10.1155/2016/3965864

**Published:** 2016-09-28

**Authors:** Yong Xie, Yi Zhang, Zebin Guo, Hongliang Zeng, Baodong Zheng

**Affiliations:** ^1^College of Pharmacy, Fujian University of Traditional Chinese Medicine, Fuzhou 350122, China; ^2^College of Food Science and Technology, Fujian Agriculture and Forestry University, Fuzhou 350002, China

## Abstract

This study aimed to investigate the effects of total alkaloids from Nelumbinis Plumula (NPA) on insulin resistance (IR) of high-fat diet- (HFD-) induced nonalcoholic fatty liver disease (NAFLD). Rats were fed with HFD for 8 weeks to induce NAFLD. Then, the effect of NPA on ameliorating IR in HFD-induced NAFLD was evaluated. Fasting serum insulin was determined using an enzyme-linked immunosorbent assay (ELISA) kit for insulin following the manufacturer's protocol. Some inflammatory cytokines such as tumor necrosis factor alpha (TNF-*α*) and interleukin-6 (IL-6) were determined using ELISA kits to assess the inflammatory burden in rats. The results showed that HFD could induce a significant increase in blood glucose and IR in rats. However, rats treated with NPA (400 or 600 mg/kg) showed improved IR and reduction in serum inflammatory cytokines TNF-*α* and IL-6. Further investigation indicated that NPA could inhibit IR by restoring the insulin receptor substrate-1 (IRS-1) and suppressing the expression of c-Jun N-terminal kinase (JNK) phosphorylation. The present results supported the view that the pathogenesis of NAFLD was complex with inflammation, together with increasing serum glucose and IR. Also, JNK and IRS phosphorylation were suggested for their involvement in the modulating of IR during NAFLD progression. Therefore, NPA may serve as a potential natural remedy against IR in NAFLD.

## 1. Introduction

Nonalcoholic fatty liver disease (NAFLD) refers to the accumulation of hepatic steatosis not due to excess alcohol consumption. NAFLD has become a serious medical problem and an increasing threat to public health. It is estimated that approximately 20%–40% of the people in various countries have excess fat accumulation in the liver [1]. NAFLD encompasses a spectrum of disorders ranging from hepatic steatosis to nonalcoholic steatohepatitis (NASH), cirrhosis, or hepatocellular carcinoma [2]. Despite its severity and high prevalence, currently no pharmacological agent is available for treating NAFLD. Many factors including lipid metabolic disturbance, insulin resistance (IR), oxidative stress, and mitochondrial dysfunction are known to cause the occurrence and aggregation of NAFLD [3].

According to the prevailing theory of NAFLD pathogenesis, IR and lipid dysregulation are the “first hit” toward NAFLD, which may trigger following “hits” spontaneously such as oxidative stress, inflammation, necrosis, apoptosis, and fibrosis [4]. Therefore, targeting steatosis and IR in the early stage of NAFLD is extremely important for the retardation of NAFLD in both basic research and clinical trials. Although the initial accumulation of fat in the liver can occur through several mechanisms, most previously done research implicates IR as the key mechanism in primary NAFLD, leading to hepatic steatosis [5]. IR is associated with an increase in peripheral lipolysis, uptake of fatty acids by the liver, and synthesis of triglycerides, which promote the accumulation of hepatic fat [6].

Nowadays, the methods for treating NAFLD involve rational diet, exercise, and medicines including metformin, statins, and fibrates. However, these drugs have some adverse effects or contraindications, and no consensus exists on the most effective drug therapies [7]. Recently, emerging evidence suggests the possible use of herbal derivatives in treating NAFLD, such as green tea [8], garlic [9], resveratrol [10], and coffee [11] in both preclinical studies and clinical trials.

Lotus seeds have been widely consumed by people in Asia. The therapeutic hepatoprotective activity of ethanolic extracts of edible lotus leaves was studied, indicating that the hepatoprotective activity of lotus leaf extract was comparable with that of a standard treatment comprising silymarin, a known hepatoprotective drug [12]. NP is the germ of the lotus seeds, which tastes bitter and generally can be removed. A previous research demonstrated the protective effect of total alkaloid and alkaloid compounds including liensinine, isoliensinine, or neferine from NP against oxidative stress induced by tertiary-butyl hydroperoxide, suggesting their involvement in the cytoprotective effect on oxidative stress in liver [13]. Furthermore, total alkaloid and alkaloid compounds from NP displayed a significant cytoprotective effect against hepatic steatosis (data not reported). However, no papers have reported on the total alkaloid from NP against NAFLD.

Therefore, the aim of the present study was to investigate the effects of NPA on ameliorating IR in high-fat diet- (HFD-) induced nonalcoholic fatty liver disease in mice. And the possible mechanisms were also studied.

## 2. Materials and Methods

### 2.1. Reagents

HFD, composed of 5% lactalbumin, 10% cholesterol, 20% lard oil, and 1% yolk powder, was obtained from Research Diets, Inc. Serum blood glucose, free fatty acid, alanine amino transferase (ALT) and aspartate aminotransferase (AST) detection kits, and bicinchoninic acid protein assay kit were obtained from Nanjing Jiancheng Institute of Biotechnology (Nanjing, China). Phosphorylated insulin receptor substrate 1 (IRS-1) and c-Jun N-terminal kinase (JNK) antibody were purchased from Sigma Chemicals Co. The insulin enzyme immunoassay kit was purchased from Puer Biotechnology Co., Ltd. (Beijing, China). Enzyme-linked immunosorbent assay (ELISA) kits for tumor necrosis factor alpha (TNF-*α*) and interleukin-6 (IL-6) were supplied by Glory Science Co. Ltd. (TX, USA).

### 2.2. Preparation of Total Alkaloid from NP

NP was purchased from Fujian Xin Zi Jin Curative Co. Ltd. (Fuzhou, Fujian, China). NPA was prepared as described previously [13]. Briefly, the peel powder was extracted with 80% ethanol. Then, the extraction was centrifuged at 1500 g for 15 min, and the supernatant was collected. Next, 5% HCl solution was used to immerse the extract, followed by the addition of NaOH to regulate pH to 9-10. The crude sample was then extracted with chloroform using liquid-liquid separation. The collected soluble fraction from chloroform was evaporated to dryness and called NPA. The alkaloid composition of NPA was also reported in previous studies [13, 14], which indicated that 3 alkaloid components of liensinine, isoliensinine, and neferine make up the majority in NPA.

### 2.3. Animals

Male Wistar rats (180–200 g) were provided by the Experimental Animal Center of Fujian University of Traditional Chinese Medicine (Fuzhou, China). The animals were housed in a room with a controlled temperature (21 ± 3°C), relative humidity (65%–70%), and 12 h light/dark cycles. They were given ad libitum access to water. After adaptation for 1 week, the rats were used for the experiment approved by the ethical committee and the Laboratory Animal Center of Fujian University of Traditional Chinese Medicine.

The rats were randomly divided into six groups (*n* = 10). Group I (normal control) was given distilled water (10 mL/kg body weight) and normal feeding. Groups II–VI were administered with HFD feeding for 8 weeks to induce NAFLD in the animal. Among them, Group II was designed as the model control group. Groups III–V were administered with NPA (200, 400, and 600 mg/kg, resp., dissolved in water) for another 4 weeks. Group VI was given compound methionine and choline bitartrate tablets as positive control medicine. At the end of the experiment, blood samples of all rats were obtained via the abdominal vein under anesthesia after an overnight fasting. Liver sample from each rat was collected for molecular measurement and stored at −80°C for the assays.

### 2.4. Serum Biochemistry

Serum was obtained from blood after centrifugation (2500 ×g, 10 min). The serum blood glucose, free fatty acid, ALT, and AST levels of rats were detected using corresponding detection kits according to the manufacturer's protocols.

### 2.5. Assessment of the Level of Serum Insulin and IR

Fasting serum insulin was determined using ELISA kit for insulin following the manufacturer's protocol. Using an ELISA reader, the absorbance of the reaction product was read at 450 nm.

The homeostasis model assessment of IR (HOMA-IR) was calculated as [fasting blood glucose (mmol/L) × fasting serum insulin (mU/L)/22.5] [15].

### 2.6. Determination of Serum Levels of TNF-*α* and IL-6 Using ELISA Kits

Some inflammatory cytokines were determined to assess the inflammatory burden in rats. An ELISA kit for TNF-*α* or interleukin-6 (IL-6) was employed for estimating the serum levels of these parameters. The assays were performed in accordance with the manufacturer's protocol.

### 2.7. Western Blotting Assay

Western blotting assay for detecting phosphorylation of JNK or IRS-1 in liver tissue was determined according to the method described by Li et al. [16] with some modification. Briefly, total protein was obtained from the livers. Then, it was denatured, resolved with sodium sulfate poly acrylamide gel electrophoresis, and transferred onto a polyvinylidene difluoride membrane (PVDF). After blocking, the membranes were individually incubated overnight with mouse anti-JNK (1 : 700 dilution), mouse anti-p-JNK (1 : 700 dilution), mouse anti-IRS-1 (1 : 700 dilution), or mouse anti-P-IRS-1 (1 : 700 dilution), respectively, followed by washing and incubation with secondary antibodies to show the resulting bands. Parallel blotting of *β*-actin was used as the internal control.

### 2.8. Statistical Analysis

All values for each group were given as mean and standard deviation. The data were analyzed using one-way analysis of variance coupled with Duncan post hoc multiple range test at 95% confidence level using SPSS (version 16.0, Chicago, USA).

## 3. Results

### 3.1. Effects of NPA on Body Weight of Rats

Before the start of the experiment, the average body weight of each group was similar. As showed in Table 1, after feeding with HFD for 8 weeks, the body weights of rats in the NAFLD group significantly increased compared with the control group, indicating that HFD had a significant influence on body weight. However, after oral administration of NPA (400 and 600 mg/kg) for 4 weeks, the body weight of rats significantly reduced compared with the NAFLD group. Therefore, the consumption of NPA could significantly reduce the body weight of NAFLD rats.

### 3.2. Serum AST and ALT Activities

**Figure 1:**
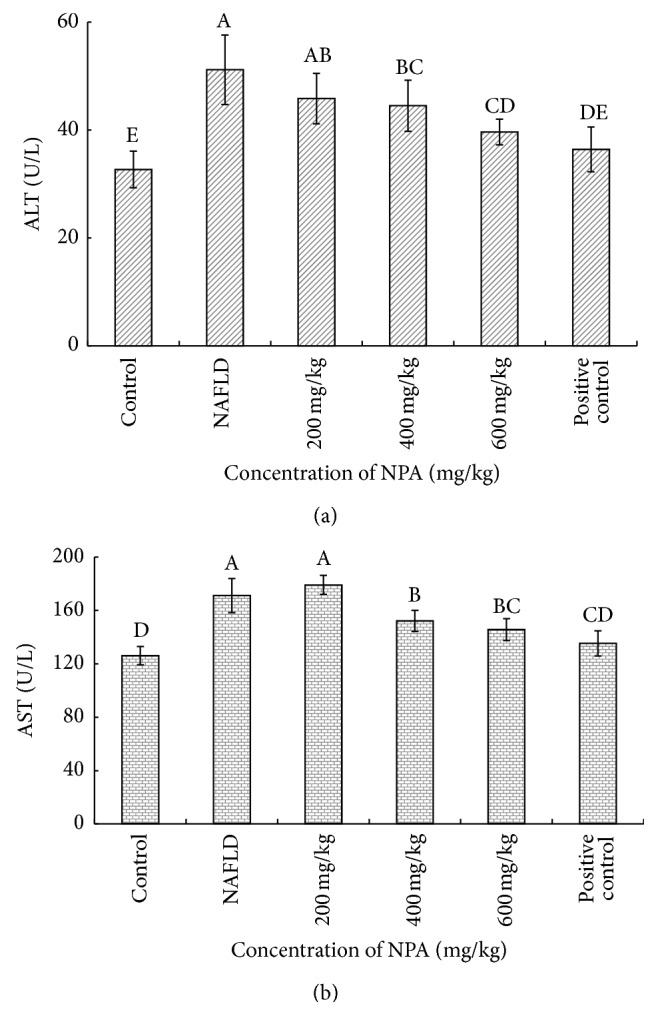
Effect of different concentrations of NPA on serum levels of ALT (a) and AST (b) in each group of rats. Different letters (A, B, C, or D) upon each column represent significant differences between the treated groups (*P* < 0.05).

Serum levels of amino transferases were indexed for general hepatic injury. To correlate hepatic injury with NAFLD, serum ALT and AST measurements were conducted in each group of rats. As shown in Figure 1, the progression of NAFLD significantly induced the higher levels of both serum ALT and AST compared with the control rats. However, the levels of ALT and AST significantly decreased in a dose-dependent manner after NPA treatment, and the hepatoprotective effects produced by NPA at the dose of 600 mg/kg reach the highest as well as the positive control group. Therefore, NPA treatment potently alleviated hepatic injury by reducing the levels of aminotransferases.

### 3.3. Effects of NPA on Serum Blood Glucose and Insulin Levels

As noted, IR and glucose metabolism dysfunction are typical clinical symptoms of NAFLD and metabolic syndrome [17]. The levels of blood glucose are given in Table 2. Eight weeks of daily ingestion of HCD contributed to the insulin resistance of the model group, which exhibited a significant increase in fasting blood glucose (FBG) compared with the control group. However, after NPA exposure, the concentrations of FBG in blood significantly decreased in a dose-dependent manner compared with the model group. These findings indicated that NPA had obvious glucose-lowering effects against NAFLD. The fasting serum insulin level increased significantly in association with the elevated FBG in the model group; however, NPA intake at higher dose significantly decreased the fasting insulin level in Groups IV-V compared with the NAFLD group.

### 3.4. HOMA-IR

A high-fat, cholesterol-rich diet induced a significant increase of IR in the NAFLD model group animals compared with the control group, as indicated by the high HOMA-IR. NPA administration in Groups IV-V could decrease their HOMA-IR level compared with the model group, reaching the control range (Table 2).

### 3.5. Effects of NPA on the Level of Serum Free Fatty Acid

**Figure 2:**
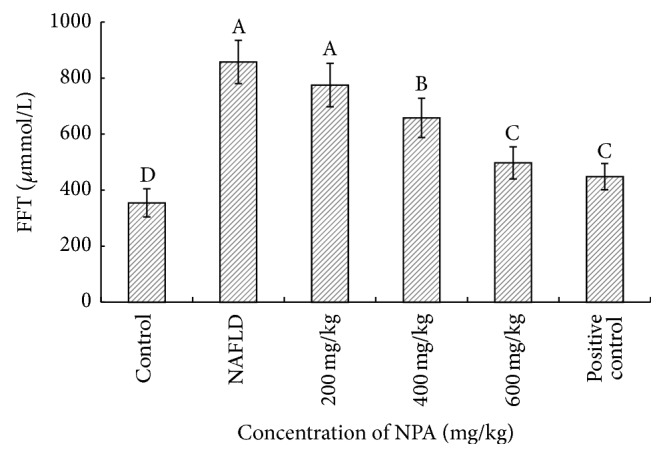
Effect of different concentrations of NPA on serum levels of FFT in each group of rats. Different letters (A, B, C, or D) upon each column represent significant differences between the treated groups (*P* < 0.05).

Our results showed that the NAFLD model rats could exhibit a much higher level of free fatty acid (FFT) compared with the control group (Figure 2). However, treating the NAFLD model with NPA at a concentration of 400 or 600 mg/kg could significantly inhibit the FFT level in serum, suggesting its significant effect on alleviating steatosis in rats.

### 3.6. Inhibitory Effects of NPA on Key Inflammatory Mediators of TNF-*α* and IL-6

**Figure 3:**
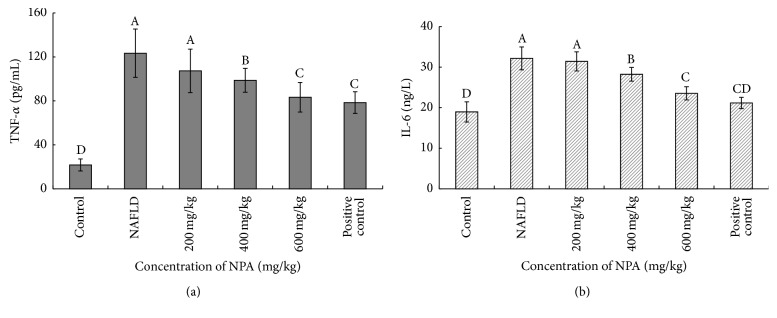
Effect of different concentrations of NPA on serum key inflammatory mediators of TNF-*α* (a) and IL-6 (b) in each group of rats. Different letters (A, B, C, or D) upon each column represent significant differences between the treated groups (*P* < 0.05).

Rats fed with HFD for 8 weeks showed a significant increase in serum TNF-*α* and IL-6 levels compared with the rats in the control group. Treatment with NPA (400 or 600 mg/kg) significantly decreased these high levels compared with the control group. Notably, NPA at a higher dose could decrease the serum IL-6 level to a much greater extent, which was comparable with the positive control group (Figures 3(a) and 3(b)).

### 3.7. JNK Phosphorylation Was Associated with IR

**Figure 4:**
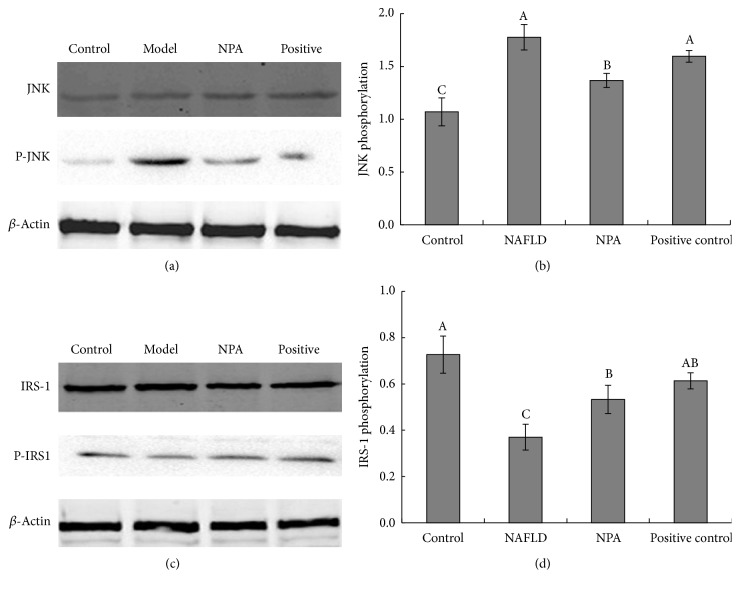
Effect of NPA on phosphorylation of JNK (a) and IRS-1 (c) in the hepatic tissue of NAPLD rats analyzed by western blot. Phosphorylation levels were evaluated by the band strength of P-JNK/JNK or P-IRS1/IRS (b and d). Liver and adipose tissue protein blots were normalized to endogenous *β*-actin. Different letters (A, B, or C) upon each column represent significant differences between the treated groups (*P* < 0.05).

The hypothesis that phosphorylated JNK is a crucial mediator for IR was tested. The results showed that JNK phosphorylation significantly increased in the NAFLD rats compared with the normal group. However, JNK phosphorylation in liver tissue could be significantly reversed by NPA treatment. Thus, increased JNK phosphorylation might explain the increased IR (Figure 4(a)).

### 3.8. Improvement in NAFLD by NPA Was Partially Attributed to the Modulation of IRS-1 Phosphorylation

IRS phosphorylation was strongly impaired in the liver of rats with NAFLD. It is well established that the insulin signaling pathway is impaired in obese objects as a result of decreased IRS phosphorylation [18]. In the present NAFLD model, western blot results showed that hepatic tissue levels of phosphorylated IRS-1 were inhibited by the induction of NAFLD. However, cotreatment with NPA completely restored the levels of these proteins (Figure 4(c)), indicating the basis of improving IR in NAFLD.

## 4. Discussion

The aim of the present study was to evaluate the hepatoprotective effect of NPA by ameliorating IR in a model of HFD-induced NAFLD. HFD is well known to increase body weight and induce IR, in addition to an inflammatory and oxidative burden in rodent models [19]. In the present study, high-fat, high-glucose diet was administered to rats to induce the occurrence of NAFLD. The aforementioned findings confirmed that 8 weeks of HFD feeding in rats resulted in increments in body weight, adiposity index, systemic IR, and inflammatory markers. HFD feeding could also increase serum FFA, causing the overloads of FFA in hepatocytes and promoting lipogenesis.

NAFLD is defined histologically as the presence of lipid droplets in more than 5% of hepatocytes, not caused by excessive alcohol consumption, drugs, or viruses. Numerous studies have demonstrated a correlation between NAFLD and IR [20]. IR represents the main link among obesity, metabolic syndrome, and liver disease with fat accumulation, that is, NAFLD [21]. Studies have also shown that hepatic steatosis may induce hepatic IR [22]. The present research investigated the effect of NPA on the metabolic alterations in the glucose level and IR in HFD-induced NAFLD. Feeding the rats with HFD resulted in dramatic increases in the FBG, FFA, and IR. The clustering of these metabolic risk factors was identified as “IR syndrome” [23].

The causes for IR during NAFLD development are numerous. It is well known that infusions of triglyceride emulsions to rapidly increase FFA levels would cause a decrease in insulin sensitivity [24]. Therefore, controlling IR, as well as associated glucose metabolism and lipid accumulation, is the first but most important step toward the therapy of NAFLD.

Fat accumulation and hepatocellular injury can lead to nonalcoholic steatohepatitis and advanced fibrosis [25]. The adipocytes are no longer considered passive cells that store excessive triglycerides but are instead considered active cells that regulate the energy balance and secrete the proinflammatory cytokines IL-6 and TNF-*α* [26]. TNF-*α* is an important mediator of IR due to its ability to influence the tyrosine kinase activity of the insulin receptor [27]. In the present study, the serum level of TNF-*α* and IL-6 increased after HFD feeding. This suggested that multiple immunomodulatory factors could contribute to the chronic inflammatory condition and hepatocytes injury observed in NAFLD. It is assumed that inflammatory factors may be mediators of IR because the plasma levels of both markers are correlated negatively with insulin sensitivity [28]. Excessive fat accumulation in hepatocytes, regardless of its cause, tends to induce the activation of nuclear factor-*κ*B, which is the key regulator of inflammation [29]. Subsequently, the expression of inflammatory cytokines TNF-*α* and IL-6 may lead to infiltration of neutrophils and inflammation in liver injury.

IR is the major abnormality in NAFLD with metabolic syndrome. Impaired IRS-1 phosphorylation is responsible for reduced insulin signaling and impaired downstream PI3K/Akt signal transduction [30]. It is well established that the insulin signaling pathway is impaired in obese patients as a result of decreased IRS phosphorylation [18]. The pathways by which increased visceral adiposity leads to IR are not fully understood. Some authors suggest that lipolysis induced by TNF-*α* and IL-6, resulting in the inhibition of IRS, may represent a major mechanism [31]. Thus, it could be considered that the restoration of IRS-1 might be beneficial for the amelioration of NAFLD. The present results showed that insulin hypersecretion was prevalent in the group of NAFLD model rats, which significantly decreased on administering NPA. Considering the survival-promoting role of IRS-1 in several hepatic diseases, particularly NAFLD, the restoration of this pathway would definitely contribute to the improvement in IR.

Lipid peroxidation products can activate transcription factor NF-kB in patients with NAFLD [32], a key transcription factor that regulates the expression of several proinflammatory cytokines and hepatic inflammation. In fact, it is now generally established that JNK1 is central to obesity-induced IR, although JNK2 might also play a contributing role [33]. The present results suggested that JNK phosphorylation might be responsible for aggravating IR, as NAFLD group advanced to more severe form. However, this progress can be reversed by administration with NPA. Hence, inhibition of JNK phosphorylation by NPA adds to its modulation effect on IR during NAPLD progression.

## 5. Conclusions

In conclusion, the present results supported the view that the pathogenesis of NAFLD was complex with inducing inflammation and IR, as well as increasing serum glucose and free fatty acid level. Administration with NPA in NAFLD rats had beneficial effects on reversing these metabolic indexes. Moreover, cotreatment with NPA could regulate IR through restoring phosphorylation of IRS-1 and alleviating phosphorylation of JNK in liver tissue, suggesting its involvement in modulating IR by protein phosphorylation during NAFLD progression. Further investigations are needed to ensure the safety, benefits, and utility of NPA in patients with NAFLD.

## Figures and Tables

**Table 1:** Body weight changes of rats during NAFLD development.

Group	Body weight at week 0 (*P* value) (g)	Body weight at week 12 (*P* value) (g)
Control	185.3 ± 6.2^a^	310.2 ± 19.1^e^
NAFLD	188.9 ± 5.1^a^	372.3 ± 22.6^a^
NAFLD + NPA (L)	190.3 ± 9.2^a^	362.0 ± 17.6^ab^
NAFLD + NPA (M)	181.5 ± 5.6^a^	347.3 ± 22.5^bc^
NAFLD + NPA (H)	180.2 ± 4.7^a^	338.8 ± 31.6^cd^
Positive control	184.1 ± 5.4^a^	322.5 ± 19.2^de^

Values are means ± SD, *n* = 10.

Control group means the normal group of rats; NAFLD group means the model group of rats; NAFLD + NPA (L) group means the NAFLD model of rats which were administrated with 200 mg/kg NPA; NAFLD + NPA (M) group means the NAFLD model of rats which were administrated with 400 mg/kg NPA; NAFLD + NPA (H) group means the NAFLD model of rats which were administrated with 600 mg/kg NPA. Different letters (a, b, c, d, or e) in the same column represent significant differences between the treated groups (*P* < 0.05).

**Table 2:** Effect of NPA on serum levels of glucose, insulin, and insulin sensitivity index in all groups of rats.

Group	FBG (*P* value) (mmol/L)	Insulin (*P* value) (mU/L)	HOMA-IR (*P* value)
Control	3.41 ± 0.37^c^	21.15 ± 3.26^d^	3.23 ± 0.75^d^
NAFLD	4.61 ± 0.52^a^	37.57 ± 3.21^a^	7.65 ± 1.34^a^
NPA (200 mg/kg)	4.34 ± 0.49^ab^	35.89 ± 5.18^a^	7.07 ± 1.45^a^
NPA (400 mg/kg)	4.12 ± 0.28^b^	30.69 ± 3.86^b^	5.60 ± 0.61^b^
NPA (600 mg/kg)	3.73 ± 0.27^c^	26.71 ± 3.11^c^	4.53 ± 0.36^c^
Positive group	3.65 ± 0.13^c^	25.58 ± 3.26^c^	4.16 ± 0.57^c^

Different letters (a, b, c, or d) in the same column represent significant differences between the treated groups (*P* < 0.05).
